# Drug and Opioid-Involved Overdose Deaths — United States, 2013–2017

**DOI:** 10.15585/mmwr.mm675152e1

**Published:** 2018-01-04

**Authors:** Lawrence Scholl, Puja Seth, Mbabazi Kariisa, Nana Wilson, Grant Baldwin

**Affiliations:** 1 Division of Unintentional Injury Prevention, National Center for Injury Prevention and Control, CDC.

The 63,632 drug overdose deaths in the United States in 2016 represented a 21.4% increase from 2015; two thirds of these deaths involved an opioid (*1*). From 2015 to 2016, drug overdose deaths increased in all drug categories examined; the largest increase occurred among deaths involving synthetic opioids other than methadone (synthetic opioids), which includes illicitly manufactured fentanyl (IMF) (*1*). Since 2013, driven largely by IMF, including fentanyl analogs (*2**–**4*), the current wave of the opioid overdose epidemic has been marked by increases in deaths involving synthetic opioids. IMF has contributed to increases in overdose deaths, with geographic differences reported (*1*). CDC examined state-level changes in death rates involving all drug overdoses in 50 states and the District of Columbia (DC) and those involving synthetic opioids in 20 states, during 2013–2017. In addition, changes in death rates from 2016 to 2017 involving all opioids and opioid subcategories,* were examined by demographics, county urbanization levels, and by 34 states and DC. Among 70,237 drug overdose deaths in 2017, 47,600 (67.8%) involved an opioid.^†^ From 2013 to 2017, drug overdose death rates increased in 35 of 50 states and DC, and significant increases in death rates involving synthetic opioids occurred in 15 of 20 states, likely driven by IMF (*2**,**3*). From 2016 to 2017, overdose deaths involving all opioids and synthetic opioids increased, but deaths involving prescription opioids and heroin remained stable. The opioid overdose epidemic continues to worsen and evolve because of the continuing increase in deaths involving synthetic opioids. Provisional data from 2018 indicate potential improvements in some drug overdose indicators;^§^ however, analysis of final data from 2018 is necessary for confirmation. More timely and comprehensive surveillance data are essential to inform efforts to prevent and respond to opioid overdoses; intensified prevention and response measures are urgently needed to curb deaths involving prescription and illicit opioids, specifically IMF.

Drug overdose deaths were identified in the National Vital Statistics System multiple cause-of-death mortality files,^¶^with death certificate data coded using the *International Classification of Diseases, Tenth Revision* (ICD-10) codes X40–44 (unintentional), X60–64 (suicide), X85 (homicide), or Y10–Y14 (undetermined intent). Among deaths with drug overdose as the underlying cause, the type of drug or drug category is indicated by the following ICD-10 multiple cause-of-death codes: opioids (T40.0, T40.1, T40.2, T40.3, T40.4, or T40.6)**; natural/semisynthetic opioids (T40.2); methadone (T40.3); heroin (T40.1); synthetic opioids other than methadone (T40.4); cocaine (T40.5); and psychostimulants with abuse potential (T43.6).^††^ Some deaths involved more than one type of drug, and these were included in rates for each drug category; thus, categories are not mutually exclusive.^§§^

Annual percent change with statistically significant trends in age-adjusted drug overdose death rates^¶¶^ for all 50 states and DC from 2013 to 2017 and in age-adjusted death rates involving synthetic opioids for 20 states that met drug specificity criteria*** were analyzed using Joinpoint regression.^†††^ Age-adjusted overdose death rates were examined from 2016 to 2017 for all opioids, prescription opioids (*5*), heroin, and synthetic opioids. Death rates were stratified by age, sex, racial/ethnic group, urbanization level,^§§§^ and state. State-level analyses included DC and 34 states with adequate drug specificity data for 2016 and 2017.^¶¶¶^ Analyses comparing changes in death rates from 2016 to 2017 used z-tests when the number of deaths was ≥100 and nonoverlapping confidence intervals based on a gamma distribution when the number was <100.****

Drug overdoses resulted in 70,237 deaths during 2017; among these, 47,600 (67.8%) involved opioids (14.9 per 100,000 population), representing a 12.0% rate increase from 2016 (Table 1). Synthetic opioids were involved in 59.8% of all opioid-involved overdose deaths; the rate increased by 45.2% from 2016 to 2017 (Table 2). From 2013 through 2017, overdose death rates increased significantly in 35 states and DC; 15 of 20 states that met drug specificity criteria had significant increases in overdose death rates involving synthetic opioids (Figure). From 2016 to 2017, death rates involving cocaine and psychostimulants increased 34.4% (from 3.2 to 4.3 per 100,000) and 33.3% (from 2.4 to 3.2 per 100,000), respectively, likely contributing to increases in drug overdose deaths; however, rates remained stable for deaths involving prescription opioids (5.2 per 100,000) (Table 1) and heroin (4.9) (Table 2).

**TABLE 1:** Annual number and age-adjusted rate of drug overdose deaths* involving all opioids^†^ and prescription opioids,^§^^,^^¶^ by sex, age, race and Hispanic origin,** urbanization level,^††^ and selected states^§§^ — United States, 2016 and 2017

Decedent characteristic	All opioids						Prescription opioids					
	2016		2017		Change from 2016 to 2017^¶¶^		2016		2017		Change from 2016 to 2017^¶¶^	
	No.	Rate	No.	Rate	Absolute rate change	% Change in rate	No.	Rate	No.	Rate	Absolute rate change	% Change in rate
**All**	**42,249**	**13.3**	**47,600**	**14.9**	1.6***	12.0***	**17,087**	**5.2**	**17,029**	**5.2**	**0.0**	**0.0**
**Sex**												
Male	28,498	18.1	32,337	20.4	2.3***	12.7***	9,978	6.2	9,873	6.1	-0.1	-1.6
Female	13,751	8.5	15,263	9.4	0.9***	10.6***	7,109	4.3	7,156	4.2	-0.1	-2.3
**Age group (yrs)**												
0–14	83	0.1	79	0.1	0.0	0.0	60	0.1	50	0.1	0.0	0.0
15–24	4,027	9.3	4,094	9.5	0.2	2.2	1,146	2.6	1,050	2.4	-0.2	-7.7
25–34	11,552	25.9	13,181	29.1	3.2***	12.4***	3,442	7.7	3,408	7.5	-0.2	-2.6
35–44	9,747	24.1	11,149	27.3	3.2***	13.3***	3,727	9.2	3,714	9.1	-0.1	-1.1
45–54	9,074	21.2	10,207	24.1	2.9***	13.7***	4,307	10.1	4,238	10.0	-0.1	-1.0
55–64	6,321	15.2	7,153	17.0	1.8***	11.8***	3,489	8.4	3,509	8.4	0.0	0.0
≥65	1,441	2.9	1,724	3.4	0.5***	17.2***	915	1.9	1,055	2.1	0.2***	10.5***
**Sex and age group (yrs)**												
Male 15–24	2,986	13.4	2,885	13.0	-0.4	-3.0	852	3.8	728	3.3	-0.5***	-13.2***
Male 25–44	15,137	35.4	17,352	40.0	4.6***	13.0***	4,527	10.6	4,516	10.4	-0.2	-1.9
Male 45–64	9,519	23.2	11,061	26.9	3.7***	15.9***	4,124	10.0	4,089	9.9	-0.1	-1.0
Female 15–24	1,041	4.9	1,209	5.7	0.8***	16.3***	294	1.4	322	1.5	0.1	7.1
Female 25–44	6,162	14.5	6,978	16.3	1.8***	12.4***	2,642	6.2	2,606	6.1	-0.1	-1.6
Female 45–64	5,876	13.6	6,299	14.6	1.0***	7.4***	3,672	8.5	3,658	8.5	0.0	0.0
Race and Hispanic origin**												
White, non-Hispanic	33,450	17.5	37,113	19.4	1.9***	10.9***	14,167	7.0	13,900	6.9	-0.1	-1.4
Black, non-Hispanic	4,374	10.3	5,513	12.9	2.6***	25.2***	1,392	3.3	1,508	3.5	0.2	6.1
Hispanic	3,440	6.1	3,932	6.8	0.7***	11.5***	1,133	2.1	1,211	2.2	0.1	4.8
American Indian/Alaska Native, non-Hispanic	369	13.9	408	15.7	1.8	12.9	173	6.5	187	7.2	0.7	10.8
Asian/Pacific Islander, non-Hispanic	323	1.5	348	1.6	0.1	6.7	131	0.7	130	0.6	-0.1	-14.3
**County urbanization level^††^**												
Large central metro	12,903	12.5	14,518	13.9	1.4***	11.2***	4,930	4.7	4,945	4.7	0.0	0.0
Large fringe metro	11,993	15.4	13,594	17.2	1.8***	11.7***	4,209	5.2	4,273	5.2	0.0	0.0
Medium metro	9,264	14.3	10,561	16.2	1.9***	13.3***	3,988	6.0	3,951	5.9	-0.1	-1.7
Small metro	3,224	11.7	3,560	12.9	1.2***	10.3***	1,471	5.2	1,479	5.2	0.0	0.0
Micropolitan (nonmetro)	3,068	12.1	3,462	13.9	1.8***	14.9***	1,475	5.7	1,440	5.6	-0.1	-1.8
Noncore (nonmetro)	1,797	10.5	1,905	11.2	0.7	6.7	1,014	5.7	941	5.3	-0.4	-7.0
**Selected states^§§^**												
**States with very good to excellent reporting (n = 27)**												
Alaska	94	12.5	102	13.9	1.4	11.2	51	6.8	51	7.0	0.2	2.9
Connecticut	855	24.5	955	27.7	3.2***	13.1***	264	7.2	273	7.7	0.5	6.9
District of Columbia	209	30.0	244	34.7	4.7	15.7	66	9.3	58	8.4	-0.9	-9.7
Georgia	918	8.8	1,014	9.7	0.9***	10.2***	536	5.1	568	5.4	0.3	5.9
Hawaii	77	5.2	53	3.4	-1.8	-34.6	55	3.6	40	2.5	-1.1	-30.6
Illinois	1,947	15.3	2,202	17.2	1.9***	12.4***	479	3.7	623	4.8	1.1***	29.7***
Iowa	183	6.2	206	6.9	0.7	11.3	92	3.1	104	3.4	0.3	9.7
Maine	301	25.2	360	29.9	4.7***	18.7***	154	12.5	100	7.6	-4.9***	-39.2***
Maryland	1,821	29.7	1,985	32.2	2.5***	8.4***	812	13.1	711	11.5	-1.6***	-12.2***
Massachusetts	1,990	29.7	1,913	28.2	-1.5	-5.1	351	4.9	321	4.6	-0.3	-6.1
Nevada	408	13.3	412	13.3	0.0	0.0	275	8.9	276	8.7	-0.2	-2.2
New Hampshire	437	35.8	424	34.0	-1.8	-5.0	89	6.5	62	4.8	-1.7	-26.2
New Mexico	349	17.5	332	16.7	-0.8	-4.6	186	9.2	171	8.5	-0.7	-7.6
New York	3,009	15.1	3,224	16.1	1.0***	6.6***	1,100	5.4	1,044	5.1	-0.3	-5.6
North Carolina	1,506	15.4	1,953	19.8	4.4***	28.6***	695	6.9	659	6.5	-0.4	-5.8
Ohio	3,613	32.9	4,293	39.2	6.3***	19.1***	867	7.7	947	8.4	0.7	9.1
Oklahoma	444	11.6	388	10.2	-1.4	-12.1	322	8.4	251	6.7	-1.7***	-20.2***
Oregon	312	7.6	344	8.1	0.5	6.6	165	3.9	154	3.5	-0.4	-10.3
Rhode Island	279	26.7	277	26.9	0.2	0.7	114	10.5	99	8.8	-1.7	-16.2
South Carolina	628	13.1	749	15.5	2.4***	18.3***	381	7.8	345	7.1	-0.7	-9.0
Tennessee	1,186	18.1	1,269	19.3	1.2	6.6	739	11.1	644	9.6	-1.5***	-13.5***
Utah	466	16.4	456	15.5	-0.9	-5.5	349	12.5	315	10.8	-1.7	-13.6
Vermont	101	18.4	114	20.0	1.6	8.7	35	5.9	40	6.3	0.4	6.8
Virginia	1,130	13.5	1,241	14.8	1.3***	9.6***	400	4.7	404	4.7	0.0	0.0
Washington	709	9.4	742	9.6	0.2	2.1	388	5.0	343	4.3	-0.7***	-14.0***
West Virginia	733	43.4	833	49.6	6.2***	14.3***	340	19.7	304	17.2	-2.5	-12.7
Wisconsin	866	15.8	926	16.9	1.1	7.0	382	6.7	362	6.4	-0.3	-4.5
**States with good reporting (n = 8)**												
Arizona	769	11.4	928	13.5	2.1***	18.4***	380	5.6	414	5.9	0.3	5.4
California	2,012	4.9	2,199	5.3	0.4***	8.2***	1,172	2.8	1,169	2.8	0.0	0.0
Colorado	536	9.5	578	10.0	0.5	5.3	258	4.5	300	5.1	0.6	13.3
Kentucky	989	23.6	1,160	27.9	4.3***	18.2***	429	10.0	433	10.2	0.2	2.0
Michigan	1,762	18.5	2,033	21.2	2.7***	14.6***	678	7.0	633	6.5	-0.5	-7.1
Minnesota	396	7.4	422	7.8	0.4	5.4	195	3.6	195	3.6	0.0	0.0
Missouri	914	15.9	952	16.5	0.6	3.8	268	4.5	253	4.1	-0.4	-8.9
Texas	1,375	4.9	1,458	5.1	0.2	4.1	617	2.2	646	2.3	0.1	4.5

**Source:** National Vital Statistics System, Mortality file.

* Deaths are classified using *the International Classification of Diseases, Tenth Revision* (ICD–10). Drug overdose deaths are identified using underlying cause-of-death codes X40–X44, X60–X64, X85, and Y10–Y14. Rates are age-adjusted using the direct method and the 2000 U.S. standard population, except for age-specific crude rates. All rates are per 100,000 population.

^†^ Drug overdose deaths, as defined, that have opium (T40.0), heroin (T40.1), natural and semisynthetic opioids (T40.2), methadone (T40.3), synthetic opioids other than methadone (T40.4), or other and unspecified narcotics (T40.6) as a contributing cause.

^§^ Drug overdose deaths, as defined, that have natural and semisynthetic opioids (T40.2) or methadone (T40.3) as a contributing cause.

^¶^ Categories of deaths are not exclusive because deaths might involve more than one drug. Summing of categories will result in more than the total number of deaths in a year.

** Data for Hispanic origin should be interpreted with caution; studies comparing Hispanic origin on death certificates and on census surveys have shown inconsistent reporting on Hispanic ethnicity. Potential race misclassification might lead to underestimates for certain categories, primarily American Indian/Alaska Native non-Hispanic and Asian/Pacific Islander non-Hispanic decedents. https://www.cdc.gov/nchs/data/series/sr_02/sr02_172.pdf.

^††^ By 2013 urbanization classification (https://www.cdc.gov/nchs/data_access/urban_rural.htm).

^§§^ Analyses were limited to states meeting the following criteria. For states with very good to excellent reporting, ≥90% of drug overdose deaths mention at least one specific drug in 2016, with the change in drug overdose deaths mentioning at least one specific drug differing by <10 percentage points from 2016 to 2017. States with good reporting had 80% to <90% of drug overdose deaths mention at least one specific drug in 2016, with the change in the percentage of drug overdose deaths mentioning at least one specific drug differing by <10 percentage points from 2016 to 2017. States included also were required to have stable rate estimates, based on ≥20 deaths, in at least two drug categories (i.e., opioids, prescription opioids, synthetic opioids other than methadone, and heroin).

^¶¶^ Absolute rate change is the difference between 2016 and 2017 rates. Percent change is the absolute rate change divided by the 2016 rate, multiplied by 100. Nonoverlapping confidence intervals based on the gamma method were used if the number of deaths was <100 in 2016 or 2017, and z-tests were used if the number of deaths was ≥100 in both 2016 and 2017.

*** Statistically significant (P-value <0.05).

**TABLE 2:** Annual number and age-adjusted rate of drug overdose deaths* involving heroin^†^ and synthetic opioids other than methadone,^§^^,^^¶^ by sex, age, race and Hispanic origin,** urbanization level,^††^ and selected states^§§^ — United States, 2016 and 2017

Decedent characteristic	Heroin						Synthetic opioids other than methadone						
	2016		2017		Change from 2016 to 2017^¶¶^		2016			2017		Change from 2016 to 2017^¶¶^	
	No.	Rate	No.	Rate	Absolute rate change	% Change in rate	No.	Rate		No.	Rate	Absolute rate change	% Change in rate
**All**	**15,469**	**4.9**	**15,482**	**4.9**	**0.0**	**0.0**	**19,413**		**6.2**	**28,466**	**9.0**	2.8***	45.2***
**Sex**													
Male	11,752	7.5	11,596	7.3	-0.2***	-2.7***	13,835		8.9	20,524	13.0	4.1***	46.1***
Female	3,717	2.4	3,886	2.5	0.1	4.2	5,578		3.5	7,942	5.0	1.5***	42.9***
**Age group (yrs)**													
0–14	^†††^	^†††^	^†††^	^†††^	^†††^	^†††^	18		^†††^	33	0.1	^†††^	^†††^
15–24	1,728	4.0	1,454	3.4	-0.6***	-15.0***	1,958		4.5	2,655	6.1	1.6***	35.6***
25–34	5,051	11.3	4,890	10.8	-0.5***	-4.4***	6,094		13.6	8,825	19.5	5.9***	43.4***
35–44	3,625	9.0	3,713	9.1	0.1	1.1	4,825		11.9	7,084	17.3	5.4***	45.4***
45–54	3,009	7.0	3,043	7.2	0.2	2.9	3,872		9.1	5,762	13.6	4.5***	49.5***
55–64	1,777	4.3	2,005	4.8	0.5***	11.6***	2,238		5.4	3,481	8.3	2.9***	53.7***
≥65	275	0.6	368	0.7	0.1***	16.7***	405		0.8	620	1.2	0.4***	50.0***
**Sex and age group (yrs)**													
Male 15–24	1,275	5.7	1,031	4.7	-1.0***	−17.5***	1,434		6.4	1,877	8.5	2.1***	32.8***
Male 25–44	6,643	15.5	6,428	14.8	-0.7***	−4.5***	8,029		18.8	11,693	27.0	8.2***	43.6***
Male 45–64	3,599	8.8	3,830	9.3	0.5***	5.7***	4,116		10.0	6,524	15.8	5.8***	58.0***
Female 15–24	453	2.1	423	2.0	-0.1	−4.8	524		2.5	778	3.7	1.2***	48.0***
Female 25–44	2,033	4.8	2,175	5.1	0.3***	6.3***	2,890		6.8	4,216	9.8	3.0***	44.1***
Female 45–64	1,187	2.8	1,218	2.8	0.0	0.0	1,994		4.6	2,719	6.3	1.7***	37.0***
Race and Hispanic origin**													
White, non-Hispanic	11,631	6.3	11,293	6.1	-0.2***	−3.2***	15,143		8.2	21,956	11.9	3.7***	45.1***
Black, non-Hispanic	1,899	4.5	2,140	4.9	0.4***	8.9***	2,391		5.6	3,832	9.0	3.4***	60.7***
Hispanic	1,555	2.8	1,669	2.9	0.1	3.6	1,505		2.7	2,152	3.7	1.0***	37.0***
American Indian/Alaska Native, non-Hispanic	131	5.0	136	5.2	0.2	4.0	113		4.1	171	6.5	2.4***	58.5***
Asian/Pacific Islander, non-Hispanic	102	0.5	119	0.5	0.0	0.0	134		0.6	189	0.8	0.2***	33.3***
**County urbanization level^††^**													
Large central metro	5,507	5.3	5,820	5.6	0.3***	5.7***	6,009		5.8	8,511	8.2	2.4***	41.4***
Large fringe metro	4,623	6.1	4,526	5.8	-0.3***	-4.9***	6,264		8.2	8,991	11.6	3.4***	41.5***
Medium metro	3,077	4.9	2,973	4.6	-0.3***	-6.1***	3,978		6.3	6,254	9.8	3.5***	55.6***
Small metro	990	3.7	972	3.6	-0.1	-2.7	1,270		4.7	1,878	7.0	2.3***	48.9***
Micropolitan (nonmetro)	860	3.6	801	3.3	-0.3	-8.3	1,228		5.0	1,860	7.7	2.7***	54.0***
Noncore (nonmetro)	412	2.6	390	2.4	-0.2	-7.7	664		4.1	972	6.0	1.9***	46.3***
**Selected states^§§^**													
**States with very good to excellent reporting (n = 27)**													
Alaska	49	6.5	36	4.9	-1.6	-24.6	^†††^		^†††^	37	4.9	^†††^	^†††^
Connecticut	450	13.1	425	12.4	-0.7	-5.3	500		14.8	686	20.3	5.5***	37.2***
District of Columbia	122	17.3	127	18.0	0.7	4.0	129		19.2	182	25.7	6.5***	33.9***
Georgia	226	2.2	263	2.6	0.4	18.2	277		2.7	419	4.1	1.4***	51.9***
Hawaii	20	1.4	10	^†††^	^†††^	^†††^	^†††^		^†††^	^†††^	^†††^	^†††^	^†††^
Illinois	1,040	8.2	1,187	9.2	1.0***	12.2***	907		7.2	1,251	9.8	2.6***	36.1***
Iowa	47	1.7	61	2.1	0.4	23.5	58		2.0	92	3.2	1.2^¶¶^	60.0^¶¶^
Maine	55	4.7	76	6.2	1.5	31.9	199		17.3	278	23.5	6.2***	35.8***
Maryland	650	10.7	522	8.6	-2.1***	-19.6***	1,091		17.8	1,542	25.2	7.4***	41.6***
Massachusetts	630	9.5	466	7.0	-2.5***	-26.3***	1,550		23.5	1,649	24.5	1.0	4.3
Nevada	86	2.9	94	3.1	0.2	6.9	53		1.7	66	2.2	0.5	29.4
New Hampshire	34	2.8	28	2.4	-0.4	-14.3	363		30.3	374	30.4	0.1	0.3
New Mexico	161	8.2	144	7.4	-0.8	-9.8	78		4.0	75	3.7	-0.3	-7.5
New York	1,307	6.5	1,356	6.8	0.3	4.6	1,641		8.3	2,238	11.3	3.0***	36.1***
North Carolina	544	5.7	537	5.6	-0.1	-1.8	601		6.2	1,285	13.2	7.0***	112.9***
Ohio	1,478	13.5	1,000	9.2	-4.3***	-31.9***	2,296		21.1	3,523	32.4	11.3***	53.6***
Oklahoma	53	1.4	61	1.6	0.2	14.3	98		2.5	102	2.6	0.1	4.0
Oregon	114	2.9	124	3.0	0.1	3.4	43		1.1	85	2.1	1.0***	90.9***
Rhode Island	25	2.5	14	^†††^	^†††^	^†††^	182		17.8	201	20.1	2.3	12.9
South Carolina	115	2.5	153	3.2	0.7	28.0	237		5.0	404	8.5	3.5***	70.0***
Tennessee	260	4.1	311	4.8	0.7	17.1	395		6.2	590	9.3	3.1***	50.0***
Utah	166	5.6	147	4.8	-0.8	-14.3	72		2.5	92	3.1	0.6	24.0
Vermont	45	8.7	41	7.3	-1.4	-16.1	53		10.1	77	13.8	3.7	36.6
Virginia	450	5.5	556	6.7	1.2***	21.8***	648		7.9	829	10.0	2.1***	26.6***
Washington	283	3.9	306	4.0	0.1	2.6	93		1.3	143	1.9	0.6***	46.2***
West Virginia	235	14.9	244	14.9	0.0	0.0	435		26.3	618	37.4	11.1***	42.2***
Wisconsin	389	7.3	414	7.8	0.5	6.8	288		5.3	466	8.6	3.3***	62.3***
**States with good reporting (n = 8)**													
Arizona	299	4.5	334	5.0	0.5	11.1	123		1.8	267	4.0	2.2***	122.2***
California	587	1.4	715	1.7	0.3***	21.4***	355		0.9	536	1.3	0.4***	44.4***
Colorado	234	4.2	224	3.9	-0.3	-7.1	72		1.3	112	2.0	0.7***	53.8***
Kentucky	311	7.6	269	6.6	-1.0	-13.2	465		11.5	780	19.1	7.6***	66.1***
Michigan	727	7.6	783	8.2	0.6	7.9	921		9.8	1,368	14.4	4.6***	46.9***
Minnesota	149	2.8	111	2.0	-0.8***	-28.6***	99		1.9	184	3.5	1.6***	84.2***
Missouri	380	6.7	299	5.3	-1.4***	-20.9***	441		7.8	618	10.9	3.1***	39.7***
Texas	530	1.9	569	2.0	0.1	5.3	250		0.9	348	1.2	0.3***	33.3***

**Source:** National Vital Statistics System, Mortality file.

* Deaths are classified using *the International Classification of Diseases, Tenth Revision* (ICD–10). Drug overdose deaths are identified using underlying cause-of-death codes X40–X44, X60–X64, X85, and Y10–Y14. Rates are age-adjusted using the direct method and the 2000 U.S. standard population, except for age-specific crude rates. All rates are per 100,000 population.

^†^ Drug overdose deaths, as defined, that have heroin (T40.1) as a contributing cause.

^§^ Drug overdose deaths, as defined, that have semisynthetic opioids other than methadone (T40.4) as a contributing cause.

^¶^ Categories of deaths are not exclusive as deaths might involve more than one drug. Summing of categories will result in more than the total number of deaths in a year.

** Data on Hispanic origin should be interpreted with caution; studies comparing Hispanic origin on death certificates and on census surveys have shown inconsistent reporting on Hispanic ethnicity. Potential race misclassification might lead to underestimates for certain categories, primarily American Indian/Alaska Native non-Hispanic and Asian/Pacific Islander non-Hispanic decedents. https://www.cdc.gov/nchs/data/series/sr_02/sr02_172.pdf.

^††^ By 2013 urbanization classification (https://www.cdc.gov/nchs/data_access/urban_rural.htm).

^§§^ Analyses were limited to states meeting the following criteria. For states with very good to excellent reporting, ≥90% of drug overdose deaths mention at least one specific drug in 2016, with the change in drug overdose deaths mentioning at least one specific drug differing by <10 percentage points from 2016 to 2017. States with good reporting had 80% to <90% of drug overdose deaths mention at least one specific drug in 2016, with the change in the percentage of drug overdose deaths mentioning at least one specific drug differing by <10 percentage points from 2016 to 2017. States included also were required to have stable rate estimates, based on ≥20 deaths, in at least two drug categories (i.e., opioids, prescription opioids, synthetic opioids other than methadone, and heroin).

^¶¶^ Absolute rate change is the difference between 2016 and 2017 rates. Percent change is the absolute rate change divided by the 2016 rate, multiplied by 100. Nonoverlapping confidence intervals based on the gamma method were used if the number of deaths was <100 in 2016 or 2017, and z-tests were used if the number of deaths was ≥100 in both 2016 and 2017. Note that the method of comparing confidence intervals is a conservative method for statistical significance; caution should be observed when interpreting a nonsignificant difference when the lower and upper limits being compared overlap only slightly. Confidence intervals of 2016 and 2017 rates of synthetic opioid-involved deaths in Iowa overlapped only slightly: (1.40, 2.39), (2.36, 3.59).

*** Statistically significant (P-value <0.05).

^†††^ Cells with ≤9 deaths are not reported. Rates based on <20 deaths are not considered reliable and are not reported.

**FIGURE:**
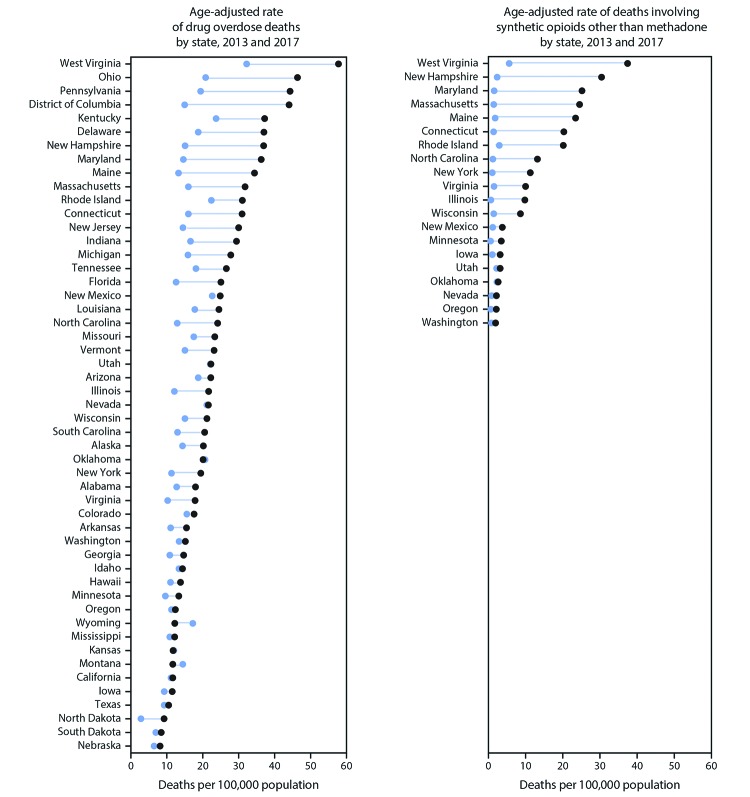
Age-adjusted rates* of drug overdose deaths and deaths involving synthetic opioids other than methadone,^†^ by state^§^ — United States, 2013 and 2017^¶^ * Rates shown are the number of deaths per 100,000 population. Age-adjusted death rates were calculated by applying age-specific death rates to the 2000 U.S. standard population age distribution. ^†^ Deaths are classified using the *International Classification of Diseases, Tenth Revision* (ICD–10). Left panel includes drug overdose deaths identified using underlying cause-of-death codes X40–X44, X60–X64, X85, and Y10–Y14. Right panel includes drug overdose deaths, as defined, that have synthetic opioids other than methadone (T40.4) as a contributing cause. ^§^ State-level analyses of overdose rates for deaths involving synthetic opioids other than methadone included 20 states that met the following criteria: 1) >80% of drug overdose death certificates named at least one specific drug in 2013–2017; 2) change from 2013 to 2017 in the percentage of death certificates reporting at least one specific drug was <10 percentage points; and 3) ≥20 deaths involving synthetic opioids other than methadone occurred each year during 2013–2017. States whose reporting of any specific drug or drugs involved in an overdose changed by ≥10 percentage points from 2013 to 2017 were excluded because drug-specific overdose numbers and rates might have changed substantially from 2013 to 2017 as a result of changes in reporting. ^¶^ Left panel: Joinpoint regression examining changes in trends from 2013 to 2017 indicated that 35 states and the District of Columbia had significant increases in drug overdose death rates from 2013 to 2017 (Alabama, Alaska, Arizona, Arkansas, Connecticut, Delaware, District of Columbia, Florida, Georgia, Hawaii, Illinois, Indiana, Iowa, Kentucky, Louisiana, Maine, Maryland, Massachusetts, Michigan, Minnesota, Missouri, New Jersey, New York, North Carolina, Ohio, Pennsylvania, Rhode Island, South Carolina, South Dakota, Tennessee, Texas, Vermont, Virginia, Washington, West Virginia, and Wisconsin). All remaining states had nonsignificant trends during this period. Right panel: Joinpoint regression examining changes in trends from 2013 to 2017 indicated that 15 states had significant increases in death rates for overdoses involving synthetic opioids other than methadone from 2013 to 2017 (Connecticut, Illinois, Iowa, Maine, Maryland, Minnesota, Nevada, New York, North Carolina, Oregon, Rhode Island, Virginia, Washington, West Virginia, and Wisconsin). The five remaining states analyzed had nonsignificant trends during this period. Significant increases in trends were not detected in some states with large absolute increases in death rates from 2013 to 2017 because of limited power to detect significant effects.

From 2016 to 2017, opioid-involved overdose deaths increased among males and females and among persons aged ≥25 years, non-Hispanic whites (whites), non-Hispanic blacks (blacks), and Hispanics (Table 1). The largest relative change occurred among blacks (25.2%), and the largest absolute rate increase was among males aged 25–44 years (an increase of 4.6 per 100,000). The largest relative change among age groups was for persons aged ≥65 years (17.2%). Counties in medium metro areas experienced the largest absolute rate increase (an increase of 1.9 per 100,000), and the largest relative rate increase occurred in micropolitan counties (14.9%). Death rates increased significantly in 15 states, with the largest relative changes in North Carolina (28.6%), Ohio (19.1%), and Maine (18.7%).

From 2016 to 2017, the prescription opioid-involved death rate decreased 13.2% among males aged 15–24 years but increased 10.5% among persons aged ≥65 years (Table 1). These death rates remained stable from 2016 to 2017 across all racial groups and urbanization levels and in most states, although five states (Maine, Maryland, Oklahoma, Tennessee, and Washington) experienced significant decreases, and one (Illinois) had a significant increase. The largest relative changes included a 29.7% increase in Illinois and a 39.2% decrease in Maine. The highest prescription opioid-involved death rates in 2017 were in West Virginia (17.2 per 100,000), Maryland (11.5), and Utah (10.8).

Heroin-involved overdose death rates declined among many groups in 2017 compared with those in 2016 (Table 2). The largest declines occurred among persons aged 15–24 years (15.0%), particularly males (17.5%), as well as in medium metro counties (6.1%). Rates declined 3.2% among whites. However, heroin-involved overdose death rates did increase among some groups; the largest relative rate increase occurred among persons aged ≥65 years (16.7%) and 55–64 years (11.6%) and among blacks (8.9%). Rates remained stable in most states, with significant decreases in five states (Maryland, Massachusetts, Minnesota, Missouri, and Ohio), and increases in three (California, Illinois, and Virginia). The largest relative decrease (31.9%) was in Ohio, and the largest relative increase (21.8%) was in Virginia. The highest heroin-involved overdose death rates in 2017 were in DC (18.0 per 100,000), West Virginia (14.9), and Connecticut (12.4).

Deaths involving synthetic opioids propelled increases from 2016 to 2017 across all demographic categories (Table 2). The highest death rate was in males aged 25–44 years (27.0 per 100,000), and the largest relative increases occurred among blacks (60.7%) and American Indian/Alaska Natives (58.5%). Deaths increased across all urbanization levels from 2016 to 2017. Twenty-three states and DC experienced significant increases in synthetic opioid-involved overdose death rates, including eight states west of the Mississippi River. The largest relative rate increase occurred in Arizona (122.2%), followed by North Carolina (112.9%) and Oregon (90.9%). The highest synthetic opioid-involved overdose death rates in 2017 were in West Virginia (37.4 per 100,000), Ohio (32.4), and New Hampshire (30.4).

## Discussion

In the United States, drug overdoses resulted in 702,568 deaths during 1999–2017, with 399,230 (56.8%) involving opioids.^††††^ From 2016 to 2017, death rates from all opioids increased, with increases driven by synthetic opioids. Deaths involving IMF have been seen primarily east of the Mississippi River;^§§§§^ however, recent increases occurred in eight states west of the Mississippi River, including Arizona, California, Colorado, Minnesota, Missouri, Oregon, Texas, and Washington.

Drug overdose death rates from 2013 to 2017 increased in most states; the influence of synthetic opioids on these rate increases was seen in approximately one quarter of all states during this same 5-year period. Overdose deaths involving cocaine and psychostimulants also have increased in recent years (*1**,**6*). Overall, the overdose epidemic continues to worsen, and it has grown increasingly complex by co-involvement of prescription and illicit drugs (*7**,**8*).^¶¶¶¶^ For example, in 2016, synthetic opioids (primarily IMF) were involved in 23.7% of deaths involving prescription opioids, 37.4% involving heroin, and 40.3% involving cocaine (*9*). In addition, death rates are increasing across multiple demographic groups. For example, although death rates involving opioids remained highest among whites, relatively large increases across several drug categories were observed among blacks.

The findings in this report are subject to at least five limitations. First, at autopsy, substances tested for vary by time and jurisdiction, and improvements in toxicologic testing might account for some reported increases. Second, the specific types of drugs involved were not included on 15% of drug overdose death certificates in 2016 and 12% in 2017, and the percentage of death certificates with at least one drug specified ranged among states from 54.7%–99.3% in 2017, limiting rate comparisons between states. Third, because heroin and morphine are metabolized similarly (*10*), some heroin deaths might have been misclassified as morphine deaths, resulting in underreporting of heroin deaths. Fourth, potential race misclassification might have led to underestimates for certain categories, primarily for American Indian/Alaska Natives and Asian/Pacific Islanders.***** Finally, most state-specific analyses were restricted to DC and a subset of states with adequate drug specificity, limiting generalizability.

Through 2017, the drug overdose epidemic continues to worsen and evolve, and the involvement of many types of drugs (e.g., opioids, cocaine, and methamphetamine) underscores the urgency to obtain more timely and local data to inform public health and public safety action. Although prescription opioid- and heroin-involved death rates were stable from 2016 to 2017, they remained high. Some preliminary indicators in 2018 point to possible improvements based on provisional data;^†††††^ however, confirmation will depend on results of pending medical investigations and analysis of final data. Overall, deaths involving synthetic opioids continue to drive increases in overdose deaths. CDC funds 32 states and DC to collect more timely and comprehensive drug overdose data, including improved toxicologic testing in opioid-involved fatal overdoses.^§§§§§^CDC is funding prevention activities in 42 states and DC.^¶¶¶¶¶^ CDC also is leveraging emergency funding to support 49 states, DC, and four territories to broaden their surveillance and response capabilities and enable comprehensive community-level responses with implementation of novel, evidence-based interventions.****** Continued efforts to ensure safe prescribing practices by following the CDC Guideline for Prescribing Opioids for Chronic Pain^††††††^ are enhanced by access to nonopioid and nonpharmacologic treatments for pain. Other important activities include increasing naloxone availability, expanding access to medication-assisted treatment, enhancing public health and public safety partnerships, and maximizing the ability of health systems to link persons to treatment and harm-reduction services.

**Summary**

**What is already known about this topic?**

The U.S. opioid overdose epidemic continues to evolve. In 2016, 66.4% of the 63,632 drug overdose deaths involved an opioid.

**What is added by this report?**

In 2017, among 70,237 drug overdose deaths, 47,600 (67.8%) involved opioids, with increases across age groups, racial/ethnic groups, county urbanization levels, and in multiple states. From 2013 to 2017, synthetic opioids contributed to increases in drug overdose death rates in several states. From 2016 to 2017, synthetic opioid-involved overdose death rates increased 45.2%.

**What are the implications for public health practice?**

Continued federal, state, and local surveillance efforts to inform evidence-based prevention, response, and treatment strategies and to strengthen public health and public safety partnerships are urgently needed.

## Footnotes

* Natural opioids include morphine and codeine, and semisynthetic opioids include drugs such as oxycodone, hydrocodone, hydromorphone, and oxymorphone. Methadone is a synthetic opioid. Prescription opioids include methadone, natural, and semisynthetic opioids. Synthetic opioids, other than methadone, include drugs such as tramadol and fentanyl. Heroin is an illicit opioid synthesized from morphine that can be a white or brown powder or a black, sticky substance.

† https://www.cdc.gov/nchs/products/databriefs/db329.htm.

§ https://www.cdc.gov/nchs/nvss/vsrr/drug-overdose-data.htm.

¶ https://www.cdc.gov/nchs/nvss/mortality_public_use_data.htm.

** T40.0 (opium) and T40.6 (other and unspecified narcotics).

†† Psychostimulants with abuse potential include drugs such as methamphetamine and 3,4-methylenedioxy-methamphetamine (MDMA).

§§ For example, a death involving both a synthetic opioid other than methadone and heroin would be included in both the synthetic opioid other than methadone and heroin death rates.

¶¶ Age-adjusted death rates were calculated by applying age-specific death rates to the 2000 U.S. Census standard population age distribution https://www.cdc.gov/nchs/data/nvsr/nvsr61/nvsr61_04.pdf.

*** State-level analyses for synthetic opioid-involved overdose deaths, comparing death rates from 2013 to 2017, included 20 states that met the following criteria: 1) >80% of drug overdose death certificates named at least one specific drug in 2013–2017; 2) change from 2013 to 2017 in the percentage of death certificates reporting at least one specific drug was <10 percentage points; and 3) ≥20 deaths involving synthetic opioids other than methadone occurred during 2013 and 2017. States whose reporting of any specific drug or drugs involved in an overdose changed by ≥10 percentage points from 2013 to 2017 were excluded because drug-specific overdose numbers and rates might have changed substantially from 2013 to 2017 as a result of changes in reporting.

††† For all analyses, a p-value of <0.05 was considered to be statistically significant. https://surveillance.cancer.gov/joinpoint/.

§§§ Categories of 2013 NCHS Urban-Rural Classification Scheme for Counties (https://www.cdc.gov/nchs/data_access/urban_rural.htm). Large central metro: Counties in metropolitan statistical areas (MSAs) of ≥1 million population that 1) contain the entire population of largest principal city of the MSA, or 2) have their entire population contained in the largest principal city of the MSA, or 3) contain at least 250,000 inhabitants of any principal city of the MSA; Large fringe metro: Counties in MSAs of ≥1 million population that did not qualify as large central metro counties; Medium metro: Counties in MSAs of populations of 250,000–999,999; Small metro: Counties in MSAs of populations <250,000; Micropolitan (nonmetropolitan counties): counties in micropolitan statistical areas; Noncore (nonmetropolitan counties): nonmetropolitan counties that did not qualify as micropolitan.

¶¶¶ State-level analyses comparing death rates from 2016 to 2017 included 34 states and DC that met the following criteria: 1) >80% of drug overdose death certificates named at least one specific drug in 2016 and 2017; 2) change from 2016 to 2017 in the percentage of death certificates reporting at least one specific drug was <10 percentage points; and 3) ≥20 deaths occurred during 2016 and 2017 in at least two opioid subcategories examined. States whose reporting of any specific drug or drugs involved in an overdose changed by ≥10 percentage points from 2016 to 2017 were excluded because drug-specific overdose numbers and rates might have changed substantially from 2016 to 2017 as a result of changes in reporting.

**** Z-tests were used if the number of deaths was ≥100, and a p-value of <0.05 was considered to be statistically significant. Nonoverlapping confidence intervals based on the gamma method were used if the number of deaths was <100 in 2015 or 2016. Note that the method of comparing confidence intervals is a conservative method for statistical significance; caution should be observed when interpreting a nonsignificant difference when the lower and upper limits being compared overlap only slightly.

†††† https://wonder.cdc.gov.

§§§§ https://emergency.cdc.gov/han/han00413.asp.

¶¶¶¶ https://www.cdc.gov/nchs/data/nvsr/nvsr67/nvsr67_09-508.pdf.

***** https://www.cdc.gov/nchs/data/series/sr_02/sr02_172.pdf.

††††† https://www.cdc.gov/nchs/nvss/vsrr/drug-overdose-data.htm.

§§§§§ https://www.cdc.gov/drugoverdose/foa/state-opioid-mm.html.

¶¶¶¶¶ https://www.cdc.gov/drugoverdose/states/state_prevention.html. https://www.cdc.gov/drugoverdose/foa/ddpi.html.

****** https://www.cdc.gov/cpr/readiness/funding-opioid.htm.

†††††† https://www.cdc.gov/drugoverdose/prescribing/guideline.html.
